# Unofficial policy: access to housing, housing information and social services among homeless drug users in Hartford, Connecticut

**DOI:** 10.1186/1747-597X-2-8

**Published:** 2007-03-07

**Authors:** Julia Dickson-Gomez, Mark Convey, Helena Hilario, A Michelle Corbett, Margaret Weeks

**Affiliations:** 1 Institute for Community Research, 2 Hartford Square West, Suite 100, Hartford, CT 06106, USA

## Abstract

**Background:**

Much research has shown that the homeless have higher rates of substance abuse problems than housed populations and that substance abuse increases individuals' vulnerability to homelessness. However, the effects of housing policies on drug users' access to housing have been understudied to date. This paper will look at the "unofficial" housing policies that affect drug users' access to housing.

**Methods:**

Qualitative interviews were conducted with 65 active users of heroin and cocaine at baseline, 3 and 6 months. Participants were purposively sampled to reflect a variety of housing statuses including homeless on the streets, in shelters, "doubled-up" with family or friends, or permanently housed in subsidized, unsubsidized or supportive housing. Key informant interviews and two focus group interviews were conducted with 15 housing caseworkers. Data were analyzed to explore the processes by which drug users receive information about different housing subsidies and welfare benefits, and their experiences in applying for these.

**Results:**

A number of unofficial policy mechanisms limit drug users' access to housing, information and services, including limited outreach to non-shelter using homeless regarding housing programs, service provider priorities, and service provider discretion in processing applications and providing services.

**Conclusion:**

Unofficial policy, i.e. the mechanisms used by caseworkers to ration scarce housing resources, is as important as official housing policies in limiting drug users' access to housing. Drug users' descriptions of their experiences working with caseworkers to obtain permanent, affordable housing, provide insights as to how access to supportive and subsidized housing can be improved for this population.

## Background

Researchers studying the causes of homelessness have frequently engaged in a polarized debate. Many have looked to the personal factors of homeless individuals as causes of homelessness [1,2]. One personal factor that has been hypothesized as a cause of homelessness is drug abuse. Research has shown that substance use problems afflict anywhere from 28 to 67% of homeless individuals [3-7] and that substance abuse increases individuals' vulnerability to homelessness [8-10]. Others have argued that structural changes, for example the loss of manufacturing jobs and affordable housing stock in inner-city neighborhoods, are the causes of the increase in homelessness over the past two decades [11,12]. More recently researchers have argued that both are important considerations. While personal characteristics, such as drug use, may not in themselves cause homelessness, they make certain individuals more vulnerable to homelessness given an increasingly competitive housing market [13-18]. Structural factors determine why pervasive homelessness exists in this historical time, while individual factors explain who is least able to compete for scarce affordable housing.

Structural factors that may contribute to drug users' greater vulnerability to homelessness include official and unofficial housing policies that determine eligibility for and access to various housing and welfare subsidies. The effects of housing policies on drug users' access to housing have been understudied to date. Official policies include the federal "One Strike and You're Out" law (P.L. 104–120, Sec.9) passed in 1996 that allows federal housing authorities to consider drug and alcohol abuse and convictions of people and their family members when making decisions to evict them from or deny access to federally subsidized housing. Many states, including Connecticut, have opted out of this law. Flat line funding of federally subsidized housing programs, such as the Housing Choice voucher program (formerly known as Section 8), and Shelter Plus Care, have limited the number of subsidies available. Both programs allow recipients to choose their own apartments on the competitive market and pay a proportion of the rent depending on recipients' income. While Connecticut does not consider drug convictions in decisions to deny applications for housing vouchers, criminalization of drug use affects drug users' access to housing in other ways, as criminal background checks are routine in many apartment rental applications. Other policies which have impacted drug users' access to housing include the Personal Responsibility and Work Opportunity Act of 1996, popularly known as Welfare Reform, in particular the elimination of the SSI Addiction Disability and a ban on receiving welfare benefits for convicted drug offenders [19-21].

Less understood are the effects of "unofficial" policy on drug users' access to housing. In this paper, unofficial policy is defined as the way in which official policy is implemented or enforced, or not, and the operating policies of organizations or individuals. This definition of unofficial policy borrows from Lipsky's [22] idea of "street level bureaucrats." For Lipsky, low-level employees who directly interact with the public, for example social workers, police officers, or unemployment counselors, ought to be viewed as policy makers rather than implementers of policy. As Lipsky puts it, the "decisions of street level bureaucrats, the routines they establish, and the devices they invent to cope with pressure, effectively become the public policy they carry out (xxii)." The pressures of work faced by street level bureaucrats include an almost infinite demand for services by the public along with inadequate resources available to workers to meet these demands. Street level bureaucrats use a number of strategies to ration services, including limiting access to information about services, creating categories of clients, exercising discretion in distributing benefits and sanctions, and increasing the costs of applying for services. Lipsky does not fully consider, however, the ways official policy may shape unofficial policy. For example, an official policy that cuts federally subsidized housing may create periods of relative scarcity, which may have direct effects on the pressures and coping mechanisms street level bureaucrats use.

Unofficial policy may help explain research that has shown that substance users are significantly less likely to exit homelessness [23] or access social services [24,25] than non-substance abusing homeless. For example, Zlotnick [23] and colleagues found that exit from homelessness was associated with greater social support and greater contact with service providers for homeless without a current substance abuse disorder, but not for homeless with current substance abuse. They suggest that this may be because substance using homeless persons may be more focused on obtaining and using drugs than gaining access to services, or that they may be unable to mobilize their social support networks. An alternative explanation, consistent with Lipsky's view of "street level bureaucrats" is that service providers may choose to devote more of their limited resources to homeless individuals without substance abuse problems whom they may see as more "deserving" or as having a greater chance at success in maintaining their housing. Supporting this second explanation is a study by Dohan and colleagues [26] that found that welfare workers generally applauded welfare reform's renewed attention to deservingness, including program emphases on client self-sufficiency and personal accountability.

Some drug users face multiple barriers to accessing and maintaining stable housing, including long-term substance abuse, mental health issues, and histories of arrest. Such individuals have been identified by researchers and advocates as "chronically homeless" [27-30]. As a result, alternatives to emergency shelters to house this population have begun to be proposed, including the Housing First Model, and supportive housing programs [30]. The Housing First model advocates for the provision of housing to drug addicted or mentally ill homeless that is not contingent on their "readiness," i.e., completing residential treatment programs or maintaining sobriety for a period of time. Rather, they advocate for housing with supportive services attached, including mental health services, addiction services, and assistance in budgeting, obtaining employment or maintaining an apartment. This is in contrast to the traditional Continuum of Care model that consists of several components including outreach, treatment and transitional housing, then supportive housing. Continuum of Care seeks to enhance clients' "housing readiness" by requiring sobriety and compliance with psychiatric treatment before placement to more permanent housing [30]. Connecticut has funded several supportive housing projects that provide affordable, service enriched rental housing for homeless and at-risk populations, many of whom are coping with mental illness, histories of substance addiction, or HIV/AIDS [31]. Some of these supportive housing programs follow the Housing First model and allow residents to choose which, if any, supportive services they wish to utilize. Other programs require residents to fulfill program requirements, such as active involvement in job training or substance abuse treatment. The effects these differing philosophies have on the ways in which service providers implement programs, i.e. the unofficial policy of these programs, has not been studied.

This paper will look at the "unofficial" policies that affect drug users' access to housing. Using longitudinal, in-depth interviews with both housed and homeless drug users and key informant interviews with housing caseworkers in Hartford, Connecticut, we will look at the process by which drug users receive information about different housing subsidies and welfare benefits, and their experiences in applying for these.

## Methods

### Design

We conducted longitudinal in-depth interviews with active drug users to explore their housing status and stability over time, and barriers and facilitators drug users face in accessing housing. Eligibility criteria included being over 18 years old and having used cocaine, crack or heroin within the last 30 days at the first interview. We sought to recruit active users of heroin and cocaine because previous research conducted by our research team indicated that these were the illicit drugs most frequently abused in Hartford, and that users of these substances had steadily increasing rates of homelessness over the past thirteen years [32-34]. Purposive sampling was used to identify and recruit drug users in various housing situations, including: 1) supportive housing, 2) subsidized housing, 3) non-subsidized housing, 4)"doubling up" with family or friends, 5) homeless in shelters, and 6) homeless on the street. We defined "doubling up" as the practice of temporarily moving in with family or friends.

In addition, we conducted key informant interviews and focus group interviews with service providers including shelter, supportive housing, and substance abuse treatment staff, and housing advocates in order to obtain service provider perspectives on the barriers and facilitators drug users face in accessing information, housing and services.

### Participants

Sixty-five drug users were interviewed at baseline. Forty-six percent of the sample was African American, 46% Puerto Rican, 8% non-Hispanic white, and 46% women. Participants were ethnically similar to other research projects conducted with active drug users in Hartford, although women were oversampled [33,35]. Fifty were located for follow-up interviews at three months. Of those who were not located, four were confirmed to be in jail, and one was confirmed to have moved out of state. Excluding those individuals who were in jail or had moved results in an overall retention rate of 83%. Forty-one were located for interviews at 6 months. Of those who were not located at 6 months, two were deceased, two were confirmed to have moved out of state, and five were in jail. Excluding those who had died, gone to jail, or moved out of state resulted in an overall retention rate of 73.2%. The refusal rate was less than 5%.

We conducted key informant interviews with six service providers including staff at three area shelters, leaders of groups advocating for low-income housing or to end homelessness, and staff at a substance abuse treatment organization. Two focus group interviews with three and four participants each were conducted with staff from an additional shelter and staff at an organization administering several supportive housing programs. These were originally designed and intended to be key informant interviews. However, staff at each organization expressed interest in being interviewed together so that they could share and compare their perspectives and experiences. Key informants and focus group participants included staff in different positions within their organizations, including the executive director of one organization, supervisors and caseworkers with more direct, daily interactions with clients. Participants were 60% female, 60% white, 30% African American, and 10% Latino. The refusal rate among service providers was approximately 50%. Most refusals were due to time constraints or scheduling problems.

### Procedure

Participant recruitment for the drug using sample was achieved through a combination of direct street recruitment and referral from other projects. For participants who were directly recruited, we targeted recruitment in locations where populations of drug users with differing housing characteristics could be found. Drug users who were homeless were recruited from each of Hartford's seven shelters or soup kitchens. Outreach staff approached potential participants in these settings, distributed HIV prevention materials such as bleach kits and condoms to initiate a general discussion about risk behaviors and assess their general eligibility for the study. Those participants who appeared interested and eligible were given an appointment card for full screening. Drug users who were doubled up with family or friends or housed in subsidized, non-subsidized or supportive housing were similarly recruited through street outreach, or from prior knowledge of their situation from ethnographic research in other research projects working with active drug users. We attempted to recruit equal numbers of drug users (approximately 10 or 11) from each of the housing statuses. In practice it was much easier to recruit participants in some housing categories than others (e.g. homeless in shelter and participants doubled up with family or friends were easier to identify and recruit than homeless on the street or drug users in supportive housing). Therefore, throughout the course of recruitment, when participants in any particular housing category became overrepresented, recruitment for that housing category was stopped and outreach and recruitment efforts focused on finding drug users in under-represented housing situations. Table 1 shows the housing status of participants at baseline, 3 and 6 months. Participants received a $25 incentive for completing each interview and a $15 bonus for completing all three interviews. Interviews were approximately 1 1/2 hours in duration. Written informed consent was obtained from all participants, both drug users and service providers, and the research protocol was approved by the Institutional Review Board at the Institute for Community Research.

**Table 1:** Frequency of Housing Statuses among Active Drug Users

	Baseline (N = 65)	3-month (N = 50)	6-month (N = 40)
Shelter	17%	28%	18%
On the street	14%	10%	5%
Double up	31%	22%	31%
Supportive	6%	6%	10%
Subsidized	15%	16%	26%
Non-subsidized	17%	18%	10%

All in-depth interview guides were project developed. Baseline interviews with drug users explored participants' housing histories over the previous two years, focusing on: reasons for moves, evictions or housing changes; types of public assistance, social services and housing subsidies applied for and accessed; the amount of time elapsed between application for housing and other social services and receipt or denial of housing or other services; and reasons given for denial of housing programs or apartment applications. To help participants construct their housing histories, we asked them to describe their current living situations and then moved back in time.

Three month follow-up interviews explored changes in housing status and access to housing programs. Baseline interviews were reviewed prior to follow-up interviews so that interview questions could be focused on participants' specific situations. If housing status changed since baseline, interviewers explored reasons for moves, eviction or housing changes, and any new applications to public assistance, social services or housing subsidies. The status or outcome of applications made or planned at baseline were explored. Six month follow-up interviews used the same interview guide as three-month interviews. Again, three-month interviews were reviewed so that questions followed up on any housing changes planned or made. Three-month interviews also included a brief quantitative survey to collect basic demographic information including age, income, length of time living in Hartford, educational level, and quantity and frequency of use of a variety of different drugs. This brief survey was added after it was determined that it was difficult to quantify such information from qualitative interviews. Six-month interviews also included brief demographic surveys that collected information on income, quantity and frequency of drug use in the last 30 days.

Service providers for key informant and focus group interviews were selected to represent a variety of organizations that may be directly or indirectly involved in assisting drug users to obtain housing. A list that included local homeless shelters, soup kitchens, drug treatment centers, mental health organizations, housing and homeless advocacy groups, and supportive housing programs, was compiled from staff knowledge, internet searches and networking with housing advocates and service providers. Potential staff members to target for interviews were also identified in an attempt to represent the ethnic and professional diversity within organizations. Project ethnographers then directly contacted staff at the organizations, explained the purpose of the study and invited staff to participate in a key informant interview, or contacted supervisors within the organization to explain the purpose of the study and ask permission to contact other staff members to participate in a key informant interview. Written informed consent was obtained from service providers. The length of interviews was 1/2 hour to 45 minutes.

Interviews with service providers focused on the facilitators and barriers that drug users face in accessing independent housing and in maintaining stable housing. We asked service providers about the characteristics of their clientele, including how clients are referred to their organization, to determine initial barriers or facilitators to accessing social or housing services for active drug users. We then asked them to describe the types of housing programs available, other services provided by the organization, and the eligibility requirements for housing and other services for their clients. We also asked them to describe the process through which they try to obtain housing or other services for their clients, the clients whom they have the most difficulty assisting in accessing housing, the strategies, if any, they use to overcome barriers in accessing housing for these difficult clients, and the clients who are the easiest to assist in accessing housing. Finally, we asked providers to describe reasons drug using clients have difficulty maintaining housing and the kinds of support services they feel are necessary to keep drug users in stable housing.

### Analysis

All interviews were tape recorded and transcribed verbatim. All text data were coded and analyzed for key themes and patterns of response using Atlas.ti software [36]. Interviews were coded for type of interview (key informant, drug user baseline, three or six month). Interviews with drug users were further coded for demographics and housing status at the time of interview. Data were then coded a first time for content. The coding tree was developed in an iterative process by the research team and applied to in-depth interviews with drug users and key-informant interviews. This first level of analysis coded for broad categories, e.g. social service application process, caseworkers, housing subsidies, shelter, or eviction. After this first level of coding, interviews were coded a second time to further refine categories and emerging themes. For example "creaming", "silting" "costs of applying for services," and "service provider discretion/priorities" were themes that emerged during this second level of coding. Excerpts presented in this paper were chosen to reflect these themes. All names of persons or organizations used in the paper are pseudonyms. Finally, in-depth interview with drug users were analyzed to capture changes over time. After all interviews that a participant had completed were coded, summaries were written for each participant that described his or her housing history, and welfare or other benefits received. Each participant's changes in housing status and the housing subsidies or other benefits applied for or received were then quantified by filling out a Housing Summary Checklist. These data were entered into SPSS and analyzed to show changes in housing status and stability, receipt of welfare or health benefits over time, and associations between housing status and applications to housing programs.

## Results

### Sample characteristics

Demographics for participants were collected at three-month interviews. Mean age of participants was 43 years (s.d. 6.8 years). Participants were low income with 63% having earned less than $500 in the last month, and 94% having earned less than $1000. At three months, 54% had smoked crack in the last 30 days, while 30% had injected heroin. Although current drug use was an eligibility criterion at baseline, many participants had entered treatment or stopped drug use by their 3-month interview. Those smoking crack at 3-months smoked a mean of 39 times in the prior 30 days (s.d. 45), while those injecting heroin at 3 months injected an average of 32 times in the prior month (s.d.3.4). Of those who completed all three interviews, 50% had moved at least once during the study period, while 20% had moved 4 or more times indicating a high degree of housing instability.

### Shelters as point of access to housing programs

In comparison with homeless participants who stayed on the street or who doubled up with family members or friends, homeless participants who stayed in shelters reported receiving more information about different housing programs available, particularly supportive housing programs, and were more likely to have applied for these and housing subsidies such as Shelter Plus Care or Section 8. Eight out of the 21 participants who stayed in a shelter at some point during the study period applied for or received supportive housing as compared to 2 out of the 27 participants who were homeless on the street or doubled up with family or friends but had not stayed in a shelter during the study period (p = .013). All the area shelters employed caseworkers whose job it is to help shelter residents access more permanent housing and other services, such as mental health or drug treatment. Shelter caseworkers are well informed of new housing programs starting in the city. Some shelters refer clients to programs run by other agencies. Other shelters have started their own supportive housing programs and so can connect their clients directly into their programs as space and funding become available. Shelter residents are often the first to hear of new housing programs, and may be able to apply and receive them before others have even heard about them.

Ethnographer: So what made you eligible to get into the [supportive housing] program at the Horizons Center? Did you have to meet some kind of criteria?

*Roger (African American, 53 years old): I was in the shelter first...I had a counselor down there that, you know, we got along real good and she said I'd be a perfect candidate for the Horizons Center. That's when they first was starting the program so they didn't have too many people that knew about it so she sent me over there, you know, and I had to go through some sort of screening*.

Supportive housing programs run through the shelter often give preferential treatment to residents of that particular shelter.

*Ralph (African American, 36): At the end of the season they [shelter staff] were talking about it [a supportive housing program], telling us about it and nobody really thought it was going to happen. But during the summer...before the [shelter] was going to open up last year, like the outreach workers, some case managers came around to all of the [other] shelters that they were at, that their clients, quote, unquote, their clients would maybe be at or maybe if they just saw us on the street they told us, "On this particular date make sure you come up the back at 9:00 in the morning. You are signing up for housing, first come, first serve."...And it was a big turnout actually*.

The shelter that was offering this particular housing program closed during the summer, so outreach workers visited other area shelters to find residents who had stayed in that shelter in the winter months. Because space and funding for these programs is limited, however, shelter caseworkers often do not do much outreach, and shelter residents may lose opportunities to apply for or receive new housing programs simply because they are not at the shelter when applications are being accepted.

*Ethnographer: What about some of the other programs that the shelters offer? Tom (African American, 53): Yeah. It's been around...Well, you know, I missed, they just had a housing program at the shelter and if you miss them, you just got to wait until it comes on the next time*.

Fifteen out of the 40 homeless participants reported that they avoid shelters, preferring to double up in other people's apartments or stay on the streets. It is not necessary to reside in a shelter to receive services from a caseworker there, and several participants who stayed in shelters reported continuing to use caseworkers in shelters after they no longer resided there. However, those who never stayed in shelters often had no experiences working with caseworkers, and therefore had very little information regarding rental subsidies or housing programs.

While 60% of the participants received state Medicaid and 93% received food stamps during the study period, putting them in regular contact with the caseworkers from the Department of Social Services, all but one reported that their state caseworkers never referred them to other organizations or departments for services to meet other needs such as housing. Rather, according to participants, their role seems to be limited to processing applications for their particular programs and ensuring that information about clients is up to date in order to determine clients' continuing eligibility.

Ethnographer: Have you ever talked to the caseworker for anything since you got your benefits?

*Dave (African American, 45): No. All they want you to do is come down every three months and fill out the paperwork so you can get it for the next three months*.

Ethnographer: Okay. So, what are your current sources of income, you're getting SSI...

*Carol (African American, 38): That's it*.

Ethnographer: So, what does he [your caseworker] do, what's your relationship like with him?

*Carol: I'm a number... That's it. I'm just a number...Let me see, my nine digit number on my card, my insurance card, that's what I am to him*.

E: What do you talk to him about?

*P: Nothing at all*.

A few participants (N = 7) reported learning about housing programs or subsidies from caseworkers at inpatient or outpatient substance abuse treatment programs, or methadone maintenance, and three reported learning about programs by word of mouth from friends or acquaintances. For the vast majority, however, shelter staff were the primary referral agents to accessing information about housing programs.

### Caseworker Priorities: "Creaming" versus "Silting"

While all shelters had full time staff dedicated to helping shelter residents obtain permanent housing, staff from different shelters or even staff within the same shelter often had differing philosophies that affected how they processed clients. Some viewed their role as "referral agent," i.e., they referred their clients to organizations administering various supportive housing programs or housing subsidies for which they might be eligible as Mrs. Roberts described in an in-depth interview.

Ethnographer: Let's say you have a client here, let's say its Project Achieve, okay, and they qualify. Do you have to do all the follow-ups?

*Mrs. Roberts: I don't, no. They do the follow-ups*.

E: Okay, they help find the apartment?

*Mrs. Roberts: I – referral. The client finds their apartment...They have a team of people, inspectors that will go out and inspect the apartment. Once I make the referral, initially I am out of it*.

Those who viewed their role as mainly referral often described a process by which they referred clients whom they thought had the best chance at success as Mrs. Roberts described.

Ethnographer: Okay. Um, could you please describe a little bit the process in which you connect the client into a program?

*Mrs. Roberts: We have applications for the different programs. If we don't, we get on the phone and call them up and ask them to send us a referral and we do referral letters. We do the screening here. If we know a person that is really actively using, as far as housing is concerned, we try to kind of get them on track because basically if they get into that apartment and they are actively using they are going to lose it. So we try not to set people up if they are*.

Lipsky (1980) described this process as "creaming" and argued that this was one way that service providers cope with the demand for services being outmatched by resources. If resources are limited, then creaming is a rational strategy to ensure that resources are not wasted. Mrs. Roberts described the greatest challenge to her job as being the magnitude of the homeless problem and the limited resources available to confront the problem.

Mrs. Roberts: The barrier is that this homeless thing had gotten so bad. It's bigger than anybody has ever imagined that it could be. You're dealing with so many people that have some type of mental illness that everybody is just overwhelmed. The money is not there. The housing is not there...

*Ethnographer: People are overworked*.

*Mrs. Roberts: Overworked, stress to no end*.

Other shelter staff described their roles as "caseworkers as advocates" actively working to increase the availability of affordable housing and particularly to provide housing to the chronically homeless. Staff of St. Mark's, a local shelter that had recently begun a supportive housing program, described this philosophy in a focus group interview.

*Carla: [In] recent years we have begun as an organization to say that providing emergency shelter is really not the solution, but housing folks. And as we learn more about the national movement and the success rates of supportive housing for folks who are addicted to drugs and alcohol and most of our folks have co-occurring disorders, a lot of mental health and drug and alcohol addictions...There is virtually no housing. You know in terms of numbers, last year we served about 1000 unduplicated individuals through our shelter and housing program. Our board last year took the bold step of saying we really are going to transition out of the business of providing emergency shelter and we are going to become a Housing First agency...to house folks who are chronically homeless...When you actually are there starting to do the work, you know with the resources to provide the subsidies and case management, "Oh my word, how do we house these folks!" We've been doing it quite truthfully with Shelter Plus Care subsidies but we don't actually control those...But then we provide the case management services to keep them housed, but now we are at a place where we are really trying to create a program with best practice methods and have a much more organized system so that we really ultimately can be a model that other folks can look at and replicate*.

Because staff at this shelter explicitly wished to serve as a positive example of the Housing First model, they engaged in what one staff member jokingly referred to as the opposite of creaming, "silting." If success can be demonstrated with even the most difficult cases, then that provides a stronger justification for increasing funding for such programs.

*Carla: Our goal has been "Let's look at the people who have the worst histories that nobody else will ever house," and that's really the approach we take, and we have created policies around that. You know we don't rule people out. We also feel strongly, and this is again the Housing First model, that you don't fix people first. They don't need to be fixed. They don't need to be ready. They just need to be housed and then you work from there and you work with intensive support*.

These differences in priorities and mandates result in differences in the ways that caseworkers assist shelter residents in obtaining housing. Whereas Mrs. Roberts described making sure that clients were "ready" for housing by referring them to substance abuse or mental health treatment programs before working with them on housing, staff at St. Mark's insisted that sobriety was not a precondition for housing. Mike who obtained housing through this program confirmed this.

Ethnographer: What did they tell you about your eligibility for the program?

*Mike (African American, 41): They told me what made me eligible was that one, I was a drug user and two, that I was chronically homeless*.

E: Okay. Meaning?

*P: Meaning that I was a prime candidate for one of the people looking for the program*.

Silting, however, may result in another bias whereby less difficult clients, i.e. those without mental illness or chronic substance abuse, are not considered "prime candidates" for the program or actively recruited. One shelter resident in fact complained that "trouble makers" had received housing through this program while he, who always followed shelter rules, had not.

### The Cost of Applying for Services

Lipsky (1980) argues that another way of rationing services is to increase the costs of applying for them. However, he also argues that increasing the cost of applying for services will only marginally affect demand, since those seeking many of the services that street level bureaucrats administer, e.g. housing subsidies, or welfare, need the services and cannot access them anywhere else. In other words, those with other options would not suffer the costs of seeking services. He argues that because of this, those seeking services from street-level bureaucrats should be considered "involuntary clients."

Shelter residents could be considered involuntary clients because they have few options to obtain affordable, permanent housing other than through housing subsidies or supportive housing programs. However, many participants in this project described weighing the costs of applying to various programs with the benefits they expected or hoped to receive. Whether or not a participant decided to apply for supportive housing or housing subsidies depended in part on their felt need. Those who were doubled-up with family members or friends, in addition to receiving less information about services, also may not have been as inclined to seek out information or apply for programs because their felt need was not as great, as described by Don who usually stayed in his girlfriend's apartment but occasionally stayed in a shelter when he had conflicts with her. He felt like he could use Section 8 because he often had difficulty paying his rent but had never applied.

Ethnographer: Let's say for example when you went to Green Shelter, did they ever talk to you about applying [for Section 8]? Did you ever apply?

*Don (African American, 45): Nope. They never, those people never explained anything like that to you. I guess there's so many people coming through that they don't have time unless you go and want to have a session with them, you know, like me and you talking, and then you might find out some of that, but I didn't never get into that because I wasn't going to be following through on it because I was going home. I was just, you know, cooling off*.

Other shelter residents chose not to apply because their expectations of receiving any benefits were small.

Ethnographer: Yeah, so you saw a lot of...hopelessness in the shelter?

*Cindy (African American, 50): Yes, I mean, it's really, really hopeless. They feel like they have no one to help them, you know, and the people [shelter staff] that are there, they're just there for the paycheck, you know... It's like a revolving wheel that there's nothing happens, you know? You just push the paper, keep pushing the paper and people's lives...you know, it's really messed up*.

Others weighed the costs of applying for housing against indignities suffered at the hands of social service or shelter staff.

Ethnographer: How's your relationship with your state worker?

*Shawn (African American, 42): She's a smart ass... [b]ecause...you try to talk nice to her, she make you feel like you're an asshole for trying to be nice... She...act like....she giving it to me out of her pockets, which she is, which you all are, but she make you feel like you beneath*.

Other costs include providing the paperwork that is required to apply for the programs or subsidies that can be difficult for persons who have been homeless for prolonged periods of time and may have lost many of their important documents. Other times, clients may not want to disclose some of their personal documents, such as arrest or medical records, which they find too personal to share and may question the necessity or relevance of such documents to their applications. Participants often expressed the feeling that these were capricious demands of caseworkers in order to delay or deny them access to housing programs.

*Alex (Puerto Rican, 37): I tried all of them and there's only one of them that tried to help me, was St. Marks [shelter] but...She got her head up her butt right now. Not like that, that's how I feel. I went to go ask her [about an application to a supportive housing program] and she kept beating around the bush telling me... I have to do a certain amount of things. I have to be on a program of some type. I told her I'm on a Methadone program, that's why I'm ill, exhausted because the Methadone gets me tired. So I'm on Methadone then she came out with something else, oh this and that and this. I said, "If it ain't one thing, it's another thing." You know, she said, "Get this and now get this." And, I ain't want to get my police report*.

Ethnographer: She needs the police report?

*Alex: Yeah*.

Ethnographer: What for?

Alex: Shelter Plus Care. That don't make no sense, huh?

Alex also implied that the program requirements, being in a drug treatment program, were too demanding for him. This sentiment was also express by Jennifer (white, 40 years old) who had applied but was not yet receiving Shelter Plus Care. She still fulfilled the supportive housing program requirements, however, in the hope that she would soon attain housing through the program. Her boyfriend, with whom she lived off and on, received the subsidy and she described the continuing demands of the program on his time.

*I guess people just don't understand when you put, when you get this certificate, this, you know, Shelter Plus Care, you have so much to do. I don't know how anybody could have a job and go to all these meetings and meet with all these people that you have to meet with every day and do everything they requiring you to do. It's just, it's a lot*.

Jennifer described having to meet with a caseworker monthly, be involved in daily sessions for her drug treatment, and attend weekly job readiness trainings. This particular program seems to have followed the Continuum of Care model, in that participating in several supportive services was mandatory and that applicants were expected to begin their participation in supportive services before they actually received their housing subsidy.

If the need for services is great enough, however, participants described being willing to put up with the costs of applying and expended a great deal of energy to get their needs met.

Ethnographer: Um, so you said you're applying for some housing subsidies right now?

Cindy: I'm trying to get an apartment so...

Ethnographer: Who are you working with?

*Cindy: My case manager, she's my case manager, she was put in the Oasis House for me to get in touch with and I talk to her. I was so tired of calling and don't hear nothing and I know she gets tired of me calling and not getting any information. I mean...if something come through I told her to grab it, I don't care where it's at right now. You know, I'm in between a rock and a hard place at this moment. I can't be picky*.

Long waiting lists also increase the cost of applying for housing subsidies such as Section 8. While waiting lists are determined by the amount of funding available, other practices, such as requiring that applicants make all requests in writing, increase costs even more.

*Chris (Puerto Rican, 48): I applied for Section 8 and they said I'm in list number thousand and something but that's been for years and I call them. They say they don't take information on the phone. You have to do it through correspondence and stuff, so I didn't bother, but sometimes they do send you a letter once in a great while and they tell you what number you're at and stuff*.

All but three of the participants who reported applying for Section 8 were homeless when they applied, either residing in shelters or doubled up with family members or friends. The likelihood that an applicant will still be living in the address listed on an application when correspondence is sent or subsidies become available is therefore very small, particularly since shelters place limits on the length of time a client can stay. When letters are sent to inform applicants that they have received a subsidy, they have a very limited time in which to accept the subsidy and find an apartment. If applicants do not respond because they never received the letter, they are placed again at the bottom of the waiting list, or their application is thrown out. Many participants reported applying for housing subsidies years before the baseline interview and having received no information regarding their applications since then. Many assumed that their applications were still in effect. Others found out that this was not the case only after they began working on their housing needs with caseworkers at other organizations.

*Jennifer: [The caseworker] filled in the paperwork [for Shelter Plus Care] and she took both of ours actually [mine and her boyfriend's] and when we left the shelter, we had to leave the shelter, our time was up, she didn't, she ripped them up and that was after a year and a half. And then I went to St. Mark's [different shelter] and I thought it was still in effect so I said, "We have, our paperwork is with, you know," and she called up, she said, "No, they said they ripped them up." And I said, "What?"..."They ripped them up? It's been almost, it's been almost two years." And she said, "No, they ripped them up." So she said, "I'm sorry but we're going to have to start all over again." So, Sheila from the St. Mark's started it all over again*.

State public assistance, such as food stamps and Medicaid, also require recipients to respond to correspondence requesting updated information twice a year to determine continued eligibility. Again, participants without a permanent address reported that they often did not receive their correspondence and frequently had their food stamps and medical benefits cut off temporarily until they could fill out the paper work and get them reinstated. As this could take between one to several weeks, it worsened their already precarious economic situation and decreased their chances of obtaining or maintaining stable housing.

### Distributing Benefits and Sanctions: Favoritism versus Discretion

Lipsky (1980) argues that while eligibility for public service benefits often may seem cut-and-dried, a considerable part of eligibility depends on the service providers' discretion. He further argues that the assignment of benefits or sanctions to clients is negotiated through "interpersonal strategies and implicit maneuvering." This negotiation, however, occurs in a context in which the service provider has much greater power over the definition of the situation and control over its outcome than the person seeking services.

Nine of the participants who stayed in shelters complained of "favoritism" in terms of who gets a bed at the shelter, how long they are allowed to stay, and who gets help with services. Sometimes this favoritism seemed based on past personal relationships that staff had with residents.

*Jose (Puerto Rican, 42): Well the staff, you could have five staff members and I don't know how they do it. They tweak allotment, but there's supposed to be some kind of list, but I think it is favoritism, you know what I'm saying. Somebody that they like, they just give them a bed, you know what I'm saying. I don't think it's by a list. I think that it basically by who knows who*.

Ethnographer: How would you describe the staff there?

*Jose: Um, they come from my world, you know the street world, most of them. Some of them are convicts from say, you know from the same type of, how would I say it, environment I came from. Some are homeless...so they been there. Some of them are more favored towards others*.

E: What brings that favoritism about?

*Jose: I think um becomes from a staff-homeless person relationship. It could stem from knowing him before he came there and you know, that sort of thing*.

This type of favoritism was confirmed by a shelter resident who reported having benefited from it.

Ethnographer: Why were you able to stay at the [shelter] for a while?

*Dave (African American, 53): Because I really, I got along with the staff real good. Some of the staff is friends of mine, I grew up with them, you know, so they, you know I got along with them real good so I was able to stay down there. I didn't have to go out in the morning, you know, I'd stay there all day if I want to, and they let me leave on the weekends because I still was seeing my ex, so I'd go stay with her on the weekends and come back Mondays*.

Other times, participants perceived that this favoritism was based on staff members' judgment of residents' deservingness.

*Jose: Yeah. The favoritism. Cause certain individuals get everything. Like a comment that one of the staff said yesterday was pretty raw, upset me. He says,... "I look out for only the people that work*."

Shelter staff also reported sometimes extending shelter stays for certain residents, which they described as reflecting staff discretion and program flexibility to meet client needs. They reported that they based these decisions on how "compliant" clients were to their treatment plan, or how actively they were trying to work on their goals. Residents could also have their stay shortened if they failed to follow shelter rules.

*Mrs. Roberts: As long as they are working towards a goal we don't have a problem of continuing with them. It is when they are not following through on the things that they need to do that we will terminate them. Getting into a physical fight with someone would cause them to be terminated immediately...It is determined through the coordinators here. Um, we call it the coordinators team because we do work together. We seek advice from one another so that we, it won't be a one-person thing, decision, and so we do have to discuss our clients*.

*Mr. Green: Upon an individual's entry into a shelter, initially they have approximately 97 days to be in the shelter. Within those 97 days hopefully they're working with a case manager and they are compliant with the service plan, agreed upon service plan between the client and the case manager. [If] they are just about ready to receive certain entitlements or assistance with employment, whatever the case may be, there is a possibility for an extension to be given to an individual as long as they are compliant with the service plan that was agreed upon*.

Some participants reported complying with program plans in order to extend their shelter stay while waiting for more permanent housing. Others feared being kicked out of the shelter because they were not able to comply with all the requirements.

Ethnographer: So they have like a time limit for people if they want to stay there?

*Mary (white, 38): They do. Usually it is 30 days, but they have counselors there so if you are doing something, you know, about it, you know, obviously they will let you stay there. They let me stay so long because I was scheduled for a certain date to go into the Christian [drug treatment] program I was going to in New Hampshire. So that's why they let me stay and because I was in the Methadone Clinic, so I really couldn't go back to my parents, you know, because there wasn't a clinic around there*.

*Maribel (Puerto Rican, 38): Yeah, but it's just, I was just telling you, it's probably going to take me a damn year before I can be able to find a damn job. I said, "What are you crazy?" I can't wait that long, you know, because sooner or later I got to start paying rent where I'm at or they gonna kick me out, you know? And my case manager is pushing me like, I feel that...what I'm doing is not enough for her*.

Ethnographer: Does Maria offer you any suggestions on where to go or is she just...

*Maribel: Yeah. She offered me, she went with me too. She's pretty good*, you *know, in helping me out and helping me do my paperwork and something. Go here, go there, but what I mean is that, it's not enough for her...I feel like she thinks I'm out there doing whatever I want to do and not doing what I'm supposed to be doing, you know? I see her once a week, every Wednesday, which tomorrow I'll be seeing her and showing her what I have done since the last visit that I had spoke to her. I have these papers that I have to sign when I go looking for work. Anywhere I go I got to put the name, the date...of the place and try to get a signature and try to get the phone number and all this, so I can show her that I am doing this, you know?*

The decision about whether or not to extend a resident's stay at a shelter can have profound effects on whether he or she is able to access more permanent housing through subsidies or supportive housing programs. As mentioned above, most people access services through caseworkers. In addition, changing address, as residents are forced to do if their shelter stay is not extended, makes it more difficult to follow through on applications for housing subsidies.

## Conclusion

The data presented in this paper illustrate that unofficial policy is as important in understanding drug users' vulnerability to homelessness and housing instability as official policy. In addition to the limits imposed by eligibility criteria and under-funding of housing subsidies, both housing caseworkers and drug users described a number of mechanisms that limit drug users' access to housing information and services. These include limited outreach regarding housing programs and subsidies to the homeless or marginally housed who avoid shelters. In addition, caseworkers prioritized clients in order to make decisions about how best to expend their limited resources and energy, "creaming" versus "silting." Another way that caseworkers rationed services is by increasing the costs of applying for them. Drug using participants described bureaucratic red tape and being treated disrespectfully by caseworkers. Finally, housing caseworkers are able to exercise considerable discretion when processing applications and serving their clients. This discretion was perceived as "favoritism" by the drug using participants interviewed in this project, and as program flexibility by caseworkers. Housing caseworkers and advocates act as "street level bureaucrats" and have developed these mechanisms as rational ways of coping with the limited resources available to perform their jobs.

Unofficial policies that are used by caseworkers to ration scarce resources help explain the relationship between structural factors (the lack of affordable housing and under-funding of subsidized housing) and personal vulnerabilities (drug abuse, arrest and mental illness) that are alternatively hypothesized to cause homelessness. Housing caseworkers perform their jobs within the constraints of the larger socio-political context operating within the United States. The Personal Responsibility and Work Opportunity Reconciliation Act (PRWORA) and the budget flat lining or cutting of many federally funded housing programs has created an increased scarcity of resources to those whose job it is to provide permanent housing to the homeless and precariously housed urban poor. At the same time, rhetoric used to justify the imposition of severe time limits on lifetime welfare benefits focus on the pernicious effects of welfare on the individual, breeding dependency and sexual immorality, and the return to "personal responsibility" and independence that revoking benefits enforced [26,37]. Drug users are particularly vilified within this system, as they constitute the undeserving poor, who have only their selfish consumption to blame for their poverty [26] and specifically targeted in official housing policy in the "One Strike Law" that bans drug users' and their families from receiving federally subsidized housing.

The service providers interviewed in this project, and whom homeless drug users must petition in order to access housing subsidies and supportive housing programs, use these same discourses to understand the reasons poor people are seeking services, and the kind of help to which they are entitled. Some saw their jobs as reforming the individuals seeking their services by providing job training assistance, mental health or drug treatment services; at the same time they blamed some clients for their homelessness because of their drug addictions, or poor work ethics. However, the passing of PRWORA also created a drastic decrease in services as state welfare offices were expected to empty welfare rolls and return recipients to work as soon as possible. Many state welfare departments disqualified welfare recipients for relatively minor infractions [38]. Like has been seen in other states [37], Connecticut Department of Social Services (DSS) caseworkers were pressured to ration services to the greatest extent possible. In the current political climate, therefore, it is hardly surprising that only one of the participants in this project reported being referred to other needed services by their DSS caseworkers, and nearly all reported having food stamps and state medical benefits discontinued for not filling out the proper re-determination paper work.

Housing caseworkers and advocates who worked in the shelters also operated in this political context. Many described clients as compliant or non-compliant, and used their discretion in order to determine who received beds on any particular night, who would be allowed to stay for an extended period at the shelter, and to whom they would devote their energy to try to help exit homelessness. The compliant homeless included those who were actively working on their "problems" by entering drug treatment or searching for a job, while the non-compliant included those who refused to comply with shelter rules or program requirements. Homeless drug users described interactions with caseworkers in overwhelmingly negative terms. They saw caseworkers as showing favoritism to some and felt that their demands of clients were unreasonable and capricious. Similar to results in this study, research on the health and social service needs of HIV infected persons has found that these persons had overwhelmingly negative experiences with service providers and case managers [39-41].

Shelter staff are also constrained by budget cuts that limit the resources available to help their clients, but have even less control than low-level DSS or HUD employees in determining who gets access to these. Shelter caseworkers used different strategies to manage their jobs under these difficult circumstances. First, shelter staff expended little energy in outreach to homeless who slept on the street or doubled up with family and friends. This is a rational strategy considering that there were too few resources to assist even those shelter residents who actively sought their services in obtaining permanent housing. Another strategy used is similar to Lipsky's "creaming" as shelter caseworkers expended time and energy assisting clients whom they thought had the greatest likelihood of success. Those deemed as having the best chance of success were those who were employed, and therefore less likely to have been incarcerated, while those who deemed unlikely to succeed included those who were actively using drugs, or with a serious mental illness. The final strategy, following the Housing First model, was "silting," in which shelter caseworkers attempted to house those deemed to be the most difficult cases, the long term homeless including chronic substance abusers, and persons with significant histories of mental illness and incarceration. By showing the success and cost-effectiveness of housing "pathological" individuals, they resisted dominant conservative discourses and advocated for expanding services to these individuals.

There are many ways of lessening the impact of the unofficial policies that serve to limit drug users' access to housing and other services. Increasing outreach to those in need of housing services who do not reside in shelters would improve access to information and housing programs to homeless persons who avoid shelters. Other valuable changes could decrease the costs of applying for services. For example, shelter staff and DSS caseworkers could receive on-going training regarding communication skills and a "customer service" approach to clients to address the lack of respect perceived by some participants. These trainings could explore and challenge caseworkers' implicit attitudes about homeless drug users and would be particularly important for organizations whose mission is to provide supportive housing to the chronically homeless. Alternative methods for the homeless to inquire about the status of their applications for housing subsidies or welfare benefits other than by mail could further decrease the costs of applying. Finally, caseworker discretion in distributing sanctions and benefits could be minimized by formalizing criteria by which decisions are made regarding extending a shelter resident's stay or who receives housing services. Improved communication regarding decision-making criteria might decrease shelter residents' perceptions of staff favoritism.

These mechanisms, however, are unlikely to be effective without devotion of significant resources and political will to solving the housing crisis. Lipsky (1988) argues that street level bureaucrats effectively become policy makers as they implement policy. Official policy, however, imposes constraints on caseworkers' ability to perform their job by defining the amount of resources available. Housing caseworkers have little incentive or power to eliminate barriers to drug users' access to housing, information and services in the current political economy. That an alternative model exists in the Housing First Model is a hopeful sign and a challenge to dominant discourse about impoverished drug users. Such challenges need to be continued and expanded in order to find solutions for chronically homeless substance abusers.

This paper is one of the first to explore how unofficial policy limits drug users' access to housing, information and service. Qualitative research is particularly well suited to explore processes such as the mechanisms housing caseworkers and advocates use to ration services. This study is additionally strengthened by its use of in-depth interviews with a larger sample of drug users than those typically included in qualitative research, the inclusion of drug users in various housing situations, the high proportion of women, and its longitudinal design. Limitations to the study include the small number of housing service providers interviewed and the lack of inclusion of non-drug using low-income, homeless or marginally housed participants. Additional qualitative and quantitative research is needed to explore ways that unofficial policy limits drug users' and non-drug users' access to housing defined as the sources of information about, perceived eligibility, application for and receipt and denial of housing programs and subsidies, welfare and social services.

## Competing interests

The author(s) declare that they have no competing interests.

## Authors' contributions

JDG conceived and designed the project, participated in the analysis and wrote the draft of the paper. MC participated in in-depth interviews, analysis of qualitative data, and helped coordinate the project. HH participated in in-depth interviews and helped in data analysis. AMC was involved in data analysis. MW participated in the design and analysis of data. All authors read and approved the final manuscript.
